# A novel predictive method for URS and laser lithotripsy using machine learning and explainable AI: results from the FLEXOR international database

**DOI:** 10.1007/s00345-025-05551-2

**Published:** 2025-05-12

**Authors:** Carlotta Nedbal, Vineet Gauhar, Sairam Adithya, Pietro Tramanzoli, Nithesh Naik, Shilpa Gite, Het Sevalia, Daniele Castellani, Frédéric Panthier, Jeremy Y.C. Teoh, Ben H. Chew, Khi Yung Fong, Mohammed Boulmani, Nariman Gadzhiev, Abhishek Gajendra Singh, Thomas R. W. Herrmann, Olivier Traxer, Bhaskar K. Somani

**Affiliations:** 1 ASST Fatebenefratelli Sacco, Urology, Milan, Italy; 2 Endourology Section, European Association of Urology, Arnhem, The Netherlands; 3 Ng Teng Fong General Hospital, Urology, Singapore, Singapore; 4 Symbiosis Institute of Technology, Engineering, Pune India; 5 Manipal Academy of Higher Education, Engineering, Manipal, India; 6 Azienda Ospedaliero-Universitaria Ospedali Riuniti di Ancona, Polytechnic University Le Marche, Ancona, Italy; 7 Sorbonne University GRC Urolithiasis no. 20, Tenon Hospital, Paris, France; 8 PIMM, UMR 8006 CNRS-Arts et Métiers ParisTech, Paris, France; 9 Progressive Endourological Association for Research and Leading Solutions (PEARLS), Paris, France; 10 The Chinese University of Hong Kong, Urology, Hong Kong China; 11 University of British Columbia, Urology, Vancouver, Canada; 12 Yong Loo Lin School of Medicine, National University of Singapore, Urology, Singapore, Singapore; 13 Boston Scientific – Urology and Pelvic Health, Paris, France; 14 Pavlov First Saint Petersburg State Medical University, Saint Petersburg, Russia; 15 Muljibhai Patel Urological Hospital, Nadiad, Gujarat India; 16 Kantonspital Frauenfeld, Spital Thurgau AG, Frauenfeld, Switzerland; 17 University Hospital Southampton NHS Foundation Trust, Southampton, UK

**Keywords:** Ureteroscopy, Urolithiasis, Machine learning, Artificial intelligence, Explainable AI, Outcomes prediction

## Abstract

**Purpose:**

We developed Machine learning (ML) algorithms to predict ureteroscopy (URS) outcomes, offering insights into diagnosis and treatment planning, personalised care and improved clinical decision-making.

**Methods:**

FLEXOR is a large international multicentric database including 6669 patients treated with URS for urolithiasis from 2015 to 2023. Preoperative and postoperative(PO) correlations were investigated through 15 ML-trained algorithms. Outcomes included stone free status (SFS, at 3-month imaging follow up), intraoperative (PCS bleeding, ureteric/PCS injury, need for postoperative drainage) and PO complications (fever, sepsis, need for reintervention). ML was applied for the prediction, correlation and logistic regression analysis. Explainable AI emphasizes key features and their contributions to the output.

**Results:**

Extra Tree Classifier achieved the best accuracy (81%) in predicting SFS. PCS bleed was negatively linked with ‘positive urine culture‘(-0.08), ‘tamsulosin‘(-0.08), ‘stone location‘(-0.10), ‘fibre optic scope‘(-0.19), ‘Moses Fibre‘(-0.09), and ‘TFL‘(-0.09), and positively with ‘elevated creatine‘(0.25), ‘fever‘(0.11), and ‘stone diameter‘(0.21). ‘PCS injury’ and ‘ureteric injury’ both showed moderate correlation with ‘elevated creatinine‘(0.11), ‘fever‘(0.10), and ‘lower pole stone‘(0.09). ‘Tamsulosin‘(0.23) use, presence of ‘multiple‘(0.25) or ‘lower pole‘(0.25) stones, ‘reusable scope‘(0.17) and ‘Moses Fibre’(0.2546) increased the risk for PO stent, while ‘digital scope’(-0.13) or ‘TFL‘(-0.29) reduced it. ‘Preoperative fever‘(0.10), ‘positive urine culture‘(0.16), and ‘stone diameter‘(0.10) may play a role in ‘PO fever’ and ‘sepsis’. SFS was mainly influenced by ‘age‘(0.12), ‘preoperative fever‘(0.09), ‘multiple stones‘(0.15), ‘stone diameter‘(0.17), ‘Moses Fibre“(0.15) and ‘TFL‘(-0.28).

**Conclusion:**

ML is valuable tool for accurately predicting outcomes by analysing pre-existing datasets. Our model demonstrated strong performance in outcomes and risks prediction, laying the groundwork for development of accessible predictive models.

**Supplementary information:**

The online version contains supplementary material available at 10.1007/s00345-025-05551-2.

## Introduction

Urolithiasis represents a significant burden on healthcare systems worldwide, affecting millions of individuals annually, and leading to severe pain, potential renal damage, and frequent hospitalizations [1]. Ureteroscopy and laser lithotripsy (fURSL) are among the most effective minimally invasive surgical options for treating urolithiasis [2]. Despite its efficacy, the outcomes can vary significantly among patients due to a multitude of factors, including stone burden, location, patient anatomy, and comorbidities [3]. Moreover, the risk of complications, such as ureteral injury, infection, and incomplete stone clearance, remains a critical concern for clinicians [4].

Traditionally, the prediction of success and risks have relied on clinician expertise, patient history, and basic imaging techniques [5, 6]. However, these methods often lack the necessary precision to consistently predict outcomes, leading to variability in treatment effectiveness and patient safety [7]. As healthcare continues to embrace technological advancements, there is a growing interest in the application of Machine Learning (ML) to improve clinical decision-making [8–12]. ML, a subset of artificial intelligence (AI), involves the development of algorithms that can learn from and make predictions based on data. In recent years, ML has demonstrated remarkable potential in various medical fields, offering enhanced diagnostic accuracy, personalized treatment plans, and predictive analytics that surpass traditional methods [13–16].

In the context of urolithiasis, ML offers predictive capabilities: analysing large datasets of preoperative, intraoperative, and postoperative variables, complex patterns and interactions - often imperceptible to the human eye - can be identified [17, 18]. ML models can be trained to predict procedural outcomes, such as stone-free stetus (SFS), and to detect early signs of potential complications, thereby enabling proactive interventions that improve patient outcomes [19–21].

This paper proposes a novel predictive method that integrates ML into fURSL practice. We explore the development and validation of ML models designed to enhance outcome prediction and facilitate the early detection of complications. Our approach leverages a combination of patient-specific factors, procedural data, and advanced computational techniques to drive towards more accurate, individualized care.

## Materials and methods

### Patients selection, data collection and procedures

Data were extracted from an international multicentric database, the FLEXOR registry, analysing the practices and outcomes of 6,669 patients who underwent fURSL for urinary stones between 2015 and 2023. This dataset involved 20 centers across 15 countries, focusing on adult patients (≥ 18 years) treated for urolithiasis via fURSL, including both recurrences and first presentations, naïve or pre-stented patients, emergency and elective procedures, unilateral or bilateral urolithiasis, stones in both ureter and kidney, all stone sizes. Exclusion criteria included pediatric patients, those undergoing ureteroscopy for non-urolithiasis conditions (e.g., upper tract tumors), and individuals who declined consent for data collection. In all cases, patients received specialized preoperative counseling, and informed consent for the procedure and data usage was obtained. Suspension of oral anticoagulant therapies was evaluated for each patient and tailored according to comorbidities and local protocols. Antibiotic prophylaxis protocols were applied based on local guidelines and varied across centers; patients with a preoperative positive urine sample (MSU) were treated according to antibiograms and presented a second negative MSU before surgery.

Data collected included demographic and preoperative details such as age, sex, presentation type (acute, during follow-up, incidental), symptoms, comorbidities, stone size, total stone burden (cumulative diameter), stone count and location (upper pole (UP), middle pole (MP), lower pole (LP), renal pelvis (RP)), MSU findings, and the presence of preoperative urinary drainage. Additionally, factors like preoperative hematuria, fever, positive MSU, and use of alpha-blocker medications (e.g., Tamsulosin) were documented. Imaging prior to surgery involved plain or contrast-enhanced CT scans to assess anatomy and stone characteristics.

Surgical details recorded included anesthesia type (spinal, epidural, general), operative duration (from instrument insertion to catheter placement), tools and devices used (e.g., ureteral access sheaths, single-use/reusable scopes, digital/fiberoptic scopes, baskets), and laser-related parameters (type, MOSES technology, dusting/fragmentation mode, lasering time). The use of postoperative stents and any intraoperative complications, such as injuries to the pelvicalyceal system (PCS) or ureter, bleeding, and transfusion requirements, were also documented. Early postoperative complications, including fever, sepsis, hematuria, and pain, were tracked and classified using the Clavien-Dindo grading system. Data on hospitalization duration and readmissions were also collected.

Follow-up assessments determined SFS through local protocols (using KUB X-rays, ultrasound, or non-contrast CT scans). SFS was defined as the absence of fragments larger than 2 mm in the urinary tract.

### ML algorithms training and data analysis

The workflow was organized into multiple stages, beginning with data cleaning and preprocessing. Initially, unnecessary spaces and irrelevant characters were removed to clean the data. Subsequently, preprocessing steps were undertaken, including imputing mode values for categorical preoperative characteristics. Statistical analyses were then carried out, which involved examining correlations, calculating variance inflation factors (VIF), and performing logistic regression for all four tasks. Following this, individual ML algorithms were trained for each task, while a multitask artificial neural network (ANN) was developed to address all tasks simultaneously.

In total, sixteen ML algorithms were selected and trained on the preprocessed data for each of the four tasks. These included logistic regression, quadratic discriminant analysis, Extra Trees Classifier, AdaBoost, CatBoost Classifier, Naïve Bayes, Bagging Classifier, Gradient Boost, Extreme Gradient Boost (XGBoost), Decision Tree, K-Nearest Neighbors (KNN), Random Forest, Linear Discriminant Analysis, and Support Vector Machines (SVM) using linear, polynomial, and radial basis function (RBF) kernels. Each algorithm was trained independently to predict outcomes based on the input features associated with each task.

To address multitasking, we implemented algorithms designed to generate multiple predictions simultaneously. A multitask ANN was specifically constructed to predict all postoperative outcomes concurrently, using shared layers for initial feature extraction, followed by task-specific layers tailored to each prediction. The architecture comprised an input layer with eight neurons, a shared hidden layer containing 128 neurons activated by a rectified linear unit (ReLU) function, and four distinct output layers with one neuron each, employing sigmoid activation functions to predict individual postoperative outcomes. The performance of ML models was assessed using confusion matrix and classification report. The confusion matrix provided counts of true positives, true negatives, false positives, and false negatives, which were used to derive metrics such as accuracy, precision, recall, and the F1 score. The classification report further detailed precision and recall metrics for each class within the tasks.

Recently, emphasis on model interpretability and explainability has increased, particularly in sensitive domains like healthcare, as complex models such as ANNs, often regarded as “black boxes,” require strategies to improve transparency. To enhance interpretability, explainable AI (XAI) techniques were employed to shed light on the predictions made by the models. Explainable Tree, Shapley additive explanations (SHAP) plot, SHAP beeswarm chart and SHAP bar chart were created for all selected outcomes. These methods highlighted key features and their contributions, offering clear insights into the factors influencing the outcomes.

The following features were selected for ML training and testing:

- Inputs: Age; Sex; Preoperative (PreOp) Haematuria; PreOp Pain; PreOp elevated creatinine (S-crea); PreOp Fever; PreOp MSU; PreOp Stent; Use of Tamsulosin; Normal/abnormal anatomy of urinary tract; Number of stones; Stone diameter; Location of stone; Suction UAS; Reusable/disposable scope; Digital/fibre optic scope; Use of Moses; Use of TFL;
- Outputs: Need for postoperative (PostOp) drainage; Residual fragments (RFs); Intraoperative complications: PCS bleeding, PCS injury, Ureteric injury; PostOp complications: Fever, Sepsis; Same-day discharge; Need for reintervention.

## Results

The findings from the FLEXOR registry, conducted by the TOWER research group, have already been published [22]. Thus, only a brief overview is provided here and a complete presentation of results is provided in the supplementary materials. The complete results from ML analysis and XAI are also available in the supplementary materials.

### Outcomes from the FLEXOR registry

The study enrolled 6,669 patients internationally. Mean patients age was 49.3 years, with a M: F ratio 2:1. Most patients presented with pain and a single stone, with a mean diameter of 10.04 mm. fURSL was predominantly performed using reusable flexible ureteroscopes in 72.0% cases, and a UAS was employed in 93.2%. The Holmium: YAG laser was used in 73.1%, with the combined dusting/fragmentation technique being the most common lithotripsy method. The average operation time was 62.40 min, and the mean hospital stay 3.62 days. Postoperative complications occurred in 535 patients, including 84 cases of sepsis. At follow-up, 78.3% of patients achieved SFS. Among those with RFs, 51.5% required further intervention.

### Prediction of need for PostOp drainage

For the prediction of PostOp drainage, the best performing algorithm was the Extra Tree Classifier, with an accuracy of 80.97% and a precision of 77.70%. At correlation analysis, factors related to the need for PostOp drainage were: use of Tamsulosin, Number of stones, Stone location and diameter, PreOp elevated S-crea, PreOp pain, Use of Moses, abnormal anatomy, use of disposable or fibreoptic scope, of Moses technology and TFL. Logistic regression analysis indicated the same variables as associated with the likelihood of Need for PostOp drainage.

### Prediction of FRs

**Fig. 1:**
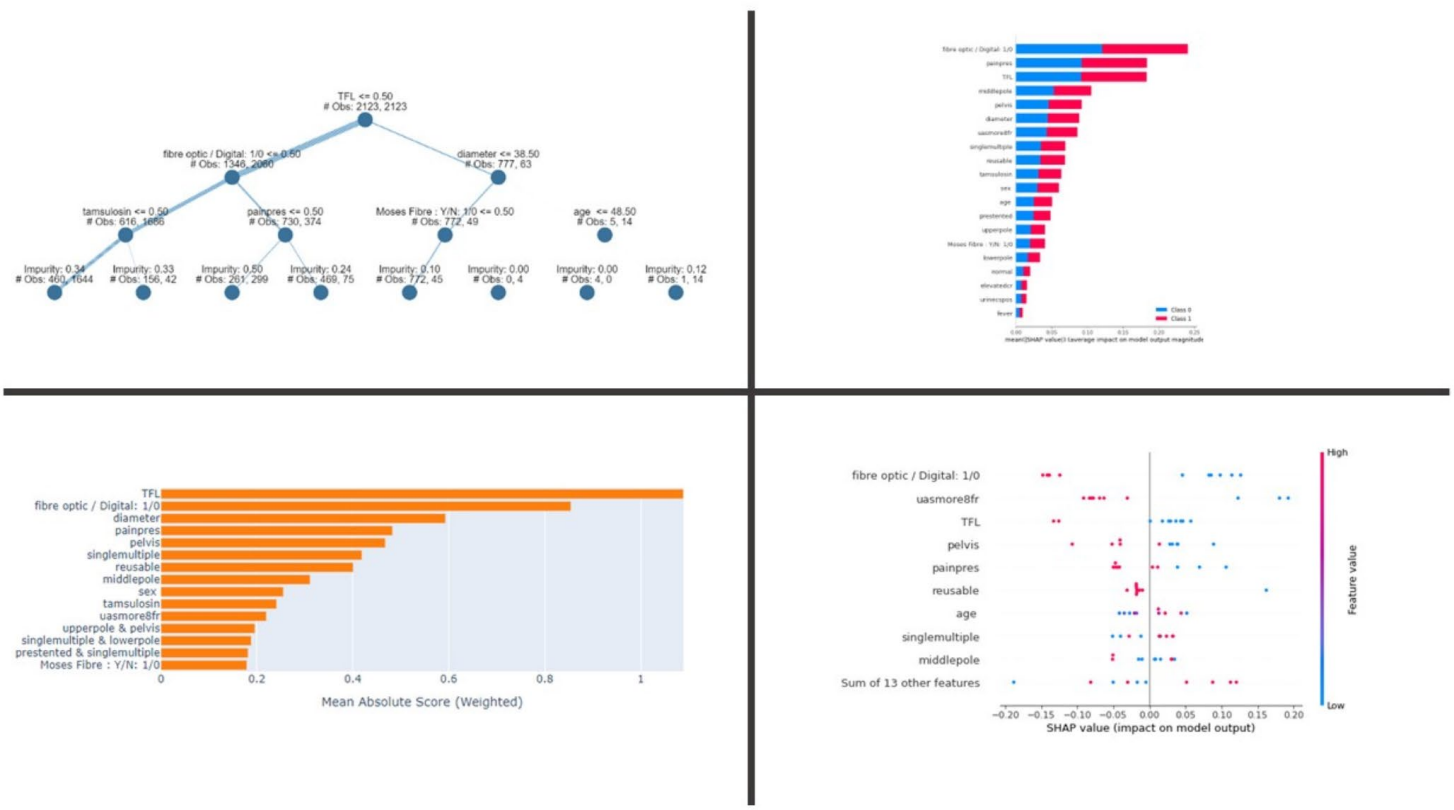
Explainable AI for RFs. Clockwise from the upper left figure: explainable tree, feature importance bar chart, shap summary plot, shap beeswarm chart

For the automated prediction of RFs, Extra Tree Classifier performed as the best algorithm, with an accuracy of 81.10% and a precision of 75.78%. Cat Boost Classifier and Random Forest followed with both an accuracy of 79.26%, and a precision of 73.59% and 73.42% respectively. Correlation analysis suggests that factors such as Age, PreOp Haematuria, PreOp elevated S-crea, PreOp Fever, Positive MSU, Number of stones, Stone diameter, Stone in LP, Use of Moses, and Use of TFL may play a role in the occurrence of FRs. Similarly, logistic regression demonstrated a significant association with Age, PreOp elevated S-crea, Number of stones, Stone diameter, Stone in RP/MP, Reusable and Fibreoptic scope, Use of Moses and TFL, Use of Tamsulosin, abnormal anatomy. XAI results (Fig. 1) demonstrated strong correlation with the use TFL and digital scopes, that seems to play a predictive role in the absence of fragments. These results were consistent in the Explainable tree, Plot Chart and SHAP Bar Chart.

### Intraoperative complications: PCS and ureteric injury

Random Forest algorithm was the best predictor of PCS injury, with 97.73% accuracy and 63.50% precision, while XGBoost achieved 96.88% accuracy and 59.27% precision in predicting ureteric injury. Explainable AI emphasized the importance of factors such as digital scope usage, TFL, stone location and higher stone burden in the risk of both PCS and ureteric injury.

Correlation analysis revealed a moderate positive correlation was observed between PCS injury and factors like elevated creatinine, fever, abnormal anatomy, multiple and larger stones, and stones in the LP/RP, with the strongest correlation seen for PCS bleed. For ureteric injury, association was noted with age and sex, preoperative haematuria/pain, positive MSU, stone diameter, number and location, elevated creatinine, fever, pre-stented patients, tamsulosin, suction UAS, reusable or fibreoptic scope, use of TFL, and Moses Fibre. Logistic regression identified key predictors of PCS injury (fever, stones in the LP/MP, and use of fibreoptic scope or TFL) and ureteric injury (elevated creatinine, fever, LP stones, and Moses Fibre).

### PostOp complications: fever and sepsis

The Extra Tree Classifier was the best ML model for predicting postoperative fever, with 91.34% accuracy and 58.20% precision; XGBoost performed slightly worse, with similar accuracy but lower precision. CatBoost Classifier was the best-performing ML algorithm in postoperative sepsis prediction, with 99.01% validation accuracy and 66.45% precision; Random Forest and Extra Tree Classifier also performed well (99.15% accuracy, 66.38% precision). Explainable AI highlighted TFL, reusable scopes, and stone characteristics (size, multiplicity, location) as key predictors of postoperative fever, while stone diameter, RP stones, and use of fibreoptic scope use emerged as key predictive features for sepsis. According to correlation analysis, preoperative MSU emerged as a key factor in postoperative fever, alongside age, sex, creatinine levels, fever, stone characteristics, and certain instrument choices. Logistic regression identified significant predictors: elevated creatinine, fever, positive MSU, tamsulosin, multiple stones, stone diameter, and MPe stones. Conversely, use of single use scopes and TFL reduced the likelihood of fever. Postoperative sepsis showed weak correlations with preoperative characteristics, and the use of TFL, suction UAS, fibreoptic scope, and Moses Fibre. Positive associations were also observed with other complications. Logistic regression highlighted significant predictors of postoperative sepsis: positive MSU, tamsulosin, increasing stone diameter, and RP stones, all increasing the odds of sepsis.

### Prediction of same-day discharge

Gradient Boost was the best-performing ML algorithm, with 83.85% validation accuracy and 76.18% precision. Cat Boost Classifier (83.66% accuracy, 75.57% precision) and XGBoost (81.53% accuracy, 72.35% precision) also performed well. Explainable AI identified type of scopes, use and type of UAS and use of tamsulosin as key predictive features, with digital scopes being the most important. Same day discharged showed weak negative correlation with patient’s demographics and preoperative features. It had a moderate negative correlation with Moses Fibre, a strong negative correlation with reusable scope, and a very strong negative correlation with fibreoptic scope. On the other hand, it has a weak positive correlation with use of TFL and suction UAS, presence of pain at presentation, fever, and stone located in UP. According to logistic regression, stone diameter, use of reusable scopes, fibreoptic scopes, Moses Fibre have negative coefficients; while use of tamsulosin and suction UAS showed positive coefficients.

### Prediction of need for reintervention

Random Forest was the best-performing ML algorithm, with 88.49% validation accuracy and 76.30% precision. XGBoost (88.21% accuracy, 75.62% precision) and Extra Tree Classifier (87.07% accuracy, 73.19% precision) also performed well. Explainable AI identified type of scopes, type of laser and stone diameters as key predictive features, with fibreoptic scopes being the most important. At correlation analysis, stone diameter, elevated creatinine, multiple stones, and stones in the RP or LP have positive correlations with reintervention. Presence of pain, patient sex, use of digital/single use scope, TFL, presence of fever, positive preoperative MSU, and use of Tamsulosin have negative correlations. Logistic regression showed negative coefficients for sex, single stone, single use, digital, Moses Fibre, and TFL have negative coefficients. Positive OR were found for elevated creatinine, fever, abnormal anatomy, stone diameter, LP stones, and use of tamsulosin.

## Discussion

This study represents the first ML-driven evaluation of operative outcomes, complications, and their correlations in a large cohort of patients undergoing fURSL for urolithiasis. 15 ML algorithms were evaluated on a substantial dataset to accurately predict complications based on preoperative and intraoperative factors, with a particular emphasis on infectious complications. We included models such as the Extra Tree Classifier, Random Forest, and CatBoost Classifier, which demonstrated high accuracy in predicting intra- and postoperative outcomes. Notably, the CatBoost Classifier achieved the highest overall performance, with 99% accuracy in predicting postoperative sepsis, offering promising potential for precise complication prediction following stone treatments. Similarly, Random Forest delivered excellent accuracy in predicting intraoperative PCS injury (97.73%) and the need for reintervention (88.49%), and XGBoost in predicting ureteric injury (96.88%). Even if with lower percentages, our ML models achieved strong accuracy in the prediction of RFs (81.10%), need for postoperative drainage (80.97%) and feasibility of same-day discharge (83.85%).

XAI techniques were also applied in this study. While being a relatively new tool, somehow still not well-known in the urological community, XAI is a powerful method to increase the transparency and interpretability of “black-box” ML models, clarifying machine-generated predictions, fostering trust in the models, and enabling informed, data-driven decisions [23]. It does not reflect correlation and regression analysis alone, but produces new insights in the analysis interpreting the standard ML results [24]. For example, the explainable decision tree revealed associations between ureteric injury and factors such as specific stone locations. Additionally, XAI tools can enhance patient consultations by visually presenting risks and personalizing treatment discussions, serving as a reference for individual risk assessments.

Our analysis revealed a significant correlation between preoperative MSU and postoperative fever and sepsis (*p* = 0.001). While the importance of performing fURS in a sterile urinary tract has been previously explored [25], this finding underscores the clinical relevance of infection control and thorough patient preparation, particularly for high-risk individuals identified through preoperative MSU. Despite tailored preoperative antibiotic therapy, patients with an initially positive urine culture remained at an elevated risk for postoperative infectious complications.

In relation to RFs and need for reintervention, our analysis revealed a strong correlation with both stone characteristics (such as diameter and position within the PCS) and instrumentation choices. The introduction of new devices like the TFL and digital scopes appears to significantly influence SFS and reintervention rates. These findings align with recent research [26, 27] and pave the way for developing ML-driven scoring systems for automated risk and outcome prediction.

While ML is a relatively novel approach in urology, prior studies have demonstrated its applicability. For instance, Vigneswaran and colleagues [28] studied the correlation between SFS and clinical and radiological factors through a ML analysis on 330 patients, revealing stone volume to be the most important factor in the prediction of SFS, with an accuracy of 74.5%. In a previous study conducted by our team on a paediatric population [29], an ML ensemble model produced high accuracy (90%) in predicting SFS, finding correlation with overall stone size, presence of multiple stones, and presence of a preoperative stent. Pietropaolo et al. [30] employed ML to predict severe sepsis following fURS in 114 patients, identifying predictors like proximal stone location, prolonged stent use, large stone size, and extended operative times, with an accuracy of 81.3%. Similarly, Castellani et al. [31] analysed data from over 1,500 patients to identify predictors of post-ureteroscopy sepsis, reporting associations with age, stone volume, and operative time, with 92% accuracy. Other research has explored ML’s potential in predicting SFS based on total stone burden or intraoperative features, further emphasizing its value in the field [32].

A notable strength of the study lies in its extensive dataset from the FLEXOR database, encompassing data from over 6,500 patients across multiple centers. This large-scale dataset enhances the robustness and generalizability of the findings. Moreover, by evaluating 15 different ML algorithms, the study identified optimal configurations that delivered exceptional performance with the top-performing models. However, the authors acknowledge several limitations, including the retrospective design, which might result in missing data, and potential biases arising from multicenter variability in procedural techniques and instrumentation. Nevertheless, these limitations arguably mirror real-world clinical practices.

Future research could focus on enhancing ML models to achieve higher precision and incorporating real-time intraoperative data to improve predictive accuracy during procedures. Long-term investigations could also assess the impact of predictive models on patient outcomes, healthcare costs, and satisfaction, further solidifying their role in clinical decision-making.

## Conclusion

Our study depicts ML as a valuable tool for the accurate prediction of outcomes by analysing pre-existing datasets, demonstrating strong performance in outcomes and risks prediction, with particular accuracy for complications. Ultimately, the integration of ML into urolithiasis treatment protocols may represent a significant step forward in achieving automatic outcome prediction and optimizing patient safety in clinical practice.

## Electronic supplementary material

Below is the link to the electronic supplementary material.

Supplementary material 1

## Data Availability

Data is provided within the manuscript or supplementary information files.
