# Supplementary material for: A novel predictive method for URS and laser lithotripsy using machine learning and explainable AI: results from the FLEXOR international database
Source: World J Urol. 2025 May 12;43(1):294. doi: 10.1007/s00345-025-05551-2 (PMC12069140; doi:10.1007/s00345-025-05551-2)
Supplement: Supplementary file 1 — Supplementary material 1 [file 345_2025_5551_MOESM1_ESM.docx]

**SUPPLEMENTARY MATERIALS**

1. **RESULTS FROM FLEXOR REGISTRY**

| **Characteristics** | **Numbers** |
| --- | --- |
| **Number of patients** | 6669 (100%) |
| **Age, years, mean (standard deviation)** | 49.34 (15.59) |
| < 40 years old | 2111 (31.6%) |
| 41–65 years old | 3494 (52.4%) |
| 66–75 years old | 731 (11.0%) |
| > 75 years old | 331 (5.0%) |
| **Gender (%)** |  |
| Male | 4407 (66.1%) |
| Female | 2262 (33.9%) |
| **Ethnicity** |  |
| Asian | 4225 (63.4%) |
| Non-Asian | 2444 (36.7%) |
| **First presentation of stone** | 5036 (75.5%) |
| **Symptoms on presentation** |  |
| Haematuria | 327 (4.9%) |
| Pain | 4178 (62.6%) |
| Elevated creatinine | 612 (9.2%) |
| Fever | 651 (9.8%) |
| Incidental Finding of Stone | 679 (10.2%) |
| Pre-stented | 3112 (46.7%) |
| Preoperative Tamsulosin Used | 1142 (17.1%) |
| **Diagnostic imaging modality** |  |
| CT Scan | 5094 (76.4%) |
| Contrast-enhanced CT scan | 814 (12.2%) |
| X-ray | 1562 (23.4%) |
| Ultrasound | 3294 (49.4%) |
| **Stone characteristics°** |  |
| HU, mean (standard deviation) | 978.94 (333.1%) |
| Multiple stones | 2732 (41.0%) |
| Size, mm, mean (standard deviation) | 10.04 (6.84) |
| Upper pole | 1474 (22.1%) |
| Mid pole | 2041 (30.6%) |
| Lower pole | 2946 (44.2%) |
| Renal pelvis | 2196 (32.9%) |
| **CT**: computed tomography, **HU**: Hounsfield Unit. | |

Table 1. FLEXOR Registry. Patients’ baseline characteristics

| **Perioperative parameters** | **Number** |
| --- | --- |
| **Preoperative antibiotics** | 5129 (76.9%) |
| **Urethral access sheath size** |  |
| Yes | 6215 (93.2%) |
| No use of sheath | 454 (6.8%) |
| **RIRS scope type** |  |
| Reusable | 4808 (72%) |
| Disposable | 1855 (27.8%) |
| **Use of Holmium Laser** | 4878 (73.1%) |
| **Use of Moses technology** | 516 (19.1%) |
| **Thulium fiber laser** | 1791 (26.9%) |
| **Lithotripsy technique** |  |
| Dusting | 3960 (59.4%) |
| Popcorning | 2337 (35%) |
| Fragmentation | 2611 (39.2%) |
| Combination | 4287 (64.3%) |
| **Laser Time, minutes, mean (standard deviation)** | 25.90 (17.76) |
| **Operation Time, minutes, mean (standard deviation)** | 62.40 (38.77) |
| **Intraoperative Complications** |  |
| PCS bleeding not requiring blood transfusion | 300 (4.5%) |
| PCS bleeding requiring blood transfusion (Clavien grade 2) | 6 (0.1%) |
| Ureteric injury due to access sheath requiring stenting (Clavien grade 3) | 119 (1.8%) |
| **URS** ureteroscopy, **Fr** French, **RIRS** retrograde intrarenal surgery, **W** Watt, **PCS** pelvicalyceal system. | |

Table 2. FLEXOR Registry. Intraoperative outcomes of 6669 cases enrolled in global FLEXOR study.

| **Characteristics** | **Numbers** |
| --- | --- |
| **Postoperative stay, days, mean (standard deviation)** | 3.62 (3.47) |
| **Day surgery** | 754 (11.3%) |
| **Overall postoperative complications** | 535 (8.0%) |
| Fever/Infections requiring antibiotics (Clavien grade 2) | 407 (6.1%) |
| Haematuria requiring blood transfusions (Clavien grade 2) | 366 (5.5%) |
| Sepsis requiring ICU admission (Clavien Grade 4) | 84 (1.3%) |
| **Post-operative imaging assessment by** |  |
| CT scan | 1748 (26.2%) |
| X-ray | 2980 (44.7%) |
| Ultrasound | 3024 (45.3%) |
| Combination | 1942 (29.1%) |
| **Residual fragments** | 1445 (21.7%) |
| **Residual fragment subsequent treatment (n = 1445)** |  |
| SWL | 257 (17.8%) |
| RIRS | 400 (27.7%) |
| PCNL | 65 (4.5%) |
| ECIRS | 22 (1.5%) |
| Observation alone | 701 (48.5%) |
| **Stone analysis** | 2845 (42.7%) |
| **ICU** intensive care unit, **CT** computed tomography, **SWL** Shock Wave Lithotripsy, **RIRS** Retrograde Intrarenal Surgery, **PCNL** percutaneous nephrolithotomy, **ECIRS** Endoscopic Combined Intrarenal Surgery | |

Table 3. FLEXOR Registry. Postoperative outcomes of 6669 cases enrolled in global FLEXOR study

1. **STATISTICAL ANALYSIS – CORRELATION ANALYSIS**

| **Variable** | **PCS bleed** | **PCS injury** | **Drainage** | **Ureteric Injury** | **Fever** | **Sepsis** | **RFs** | **Re-intervention** | **Day Surgery** |
| --- | --- | --- | --- | --- | --- | --- | --- | --- | --- |
| Age | -0.003170 | -0.004863 | 0.138228 | -0.014135 | 0.035456 | 0.031412 | 0.124823 | 0.025098 | -0.018285 |
| Sex | -0.040892 | 0.011463 | 0.036181 | 0.004986 | 0.052906 | 0.019708 | 0.004533 | -0.049636 | -0.002825 |
| Haematuria | -0.044279 | -0.022638 | 0.080924 | -0.010728 | 0.000757 | -0.016825 | 0.039428 | 0.015767 | -0.056712 |
| Pain | -0.066932 | -0.017943 | -0.148337 | -0.071161 | -0.010496 | -0.023600 | -0.196383 | -0.093162 | 0.017686 |
| Elevated S-creatinine | 0.246391 | 0.068325 | 0.176771 | 0.114368 | 0.065936 | -0.003680 | 0.075817 | 0.112204 | -0.018663 |
| Fever | 0.107065 | 0.103861 | 0.088773 | 0.098726 | 0.100775 | 0.036178 | 0.091867 | 0.063988 | 0.004308 |
| Positive MSU | -0.075773 | -0.034164 | 0.092885 | -0.008808 | 0.156843 | 0.073836 | 0.058155 | -0.049291 | -0.006421 |
| Pre-stented | 0.061773 | -0.029140 | 0.124605 | 0.033496 | 0.052945 | 0.019713 | 0.000656 | 0.001558 | 0.005445 |
| Use of tamsulosin | -0.084951 | -0.041432 | 0.231407 | 0.014848 | 0.052945 | 0.063918 | -0.108165 | -0.070476 | 0.016929 |
| Normal anatomy | 0.046825 | 0.015679 | -0.108228 | 0.011009 | -0.032321 | -0.055386 | -0.029984 | -0.001451 | -0.005495 |
| Number of stone | 0.055558 | 0.007743 | 0.246163 | 0.072390 | -0.001515 | -0.005625 | 0.152274 | 0.078915 | -0.001034 |
| Stone diameter | 0.205208 | 0.023892 | -0.092386 | -0.020274 | 0.066745 | 0.102052 | 0.169558 | 0.201387 | -0.060599 |
| Upper pole stone | -0.004478 | -0.020369 | 0.133001 | 0.010968 | 0.019191 | 0.015551 | 0.034086 | -0.000283 | 0.036433 |
| Middle pole stone | -0.104171 | -0.059587 | 0.032831 | -0.012267 | 0.034342 | 0.002102 | 0.030540 | -0.022737 | -0.006307 |
| Lower pole stone | 0.096668 | 0.083940 | 0.250458 | 0.091035 | 0.007514 | -0.005865 | 0.145266 | 0.109420 | -0.001066 |
| Pelvic stone | 0.113092 | 0.006671 | -0.101690 | 0.003689 | 0.005471 | 0.047086 | -0.068277 | 0.045917 | -0.022424 |
| Suction UAS | -0.019192 | -0.012045 | -0.069106 | -0.016245 | -0.014922 | -0.008952 | -0.036040 | -0.026371 | 0.175092 |
| Reusable scope | -0.069234 | -0.006156 | 0.165452 | -0.003275 | -0.028477 | -0.066089 | 0.026478 | -0.054525 | -0.275076 |
| Fibreoptic scope | -0.190538 | -0.094738 | -0.129643 | -0.046743 | 0.002355 | -0.040082 | -0.190240 | -0.211106 | -0.294304 |
| Moses Fibre | -0.087458 | -0.034600 | 0.254627 | 0.029864 | 0.014154 | -0.031467 | 0.154578 | 0.011109 | -0.160649 |
| TFL | -0.091666 | -0.054918 | -0.290501 | -0.042145 | -0.023731 | 0.044175 | -0.276690 | -0.155835 | 0.217401 |
| PCS bleed | 1.000000 | 0.250529 | 0.197139 | 0.146717 | 0.060196 | 0.067505 | 0.255168 | 0.327595 | -0.071500 |
| PCS injury | 0.250529 | 1.000000 | 0.074861 | 0.168057 | 0.003802 | 0.050603 | 0.097821 | 0.123861 | -0.007552 |
| Postoperative drainage | 0.197139 | 0.074861 | 1.000000 | 0.172981 | 0.079495 | 0.011296 | 0.247951 | 0.243619 | -0.135674 |
| Ureteric injury | 0.146717 | 0.168057 | 0.172981 | 1.000000 | 0.026067 | 0.032145 | 0.090794 | 0.082168 | -0.012063 |
| PostOp fever | 0.060196 | 0.003802 | 0.079495 | 0.026067 | 1.000000 | 0.208936 | 0.059260 | 0.016352 | -0.057900 |
| PostOp sepsis | 0.067505 | 0.050603 | 0.011296 | 0.032145 | 0.208936 | 1.000000 | 0.005860 | 0.005675 | 0-007434 |
| RFs | 0.255168 | 0.097821 | 0.247951 | 0.090794 | 0.059260 | 0.005860 | 1.000000 | 0.607843 | -0.022771 |
| Reintervention | 0.327595 | 0.123861 | 0.243619 | 0.082168 | 0.016352 | 0.005675 | 0.607843 | 1.000000 | -0.097500 |
| Day surgery | -0.071500 | -0.007552 | -0.135674 | -0.012063 | -0.057900 | 0.007434 | -0.022771 | -0.097500 | 1.000000 |

Table 4. Correlation analysis

1. **STATISTICAL ANALYSIS – LOGISTIC REGRESSION ANALYSIS**


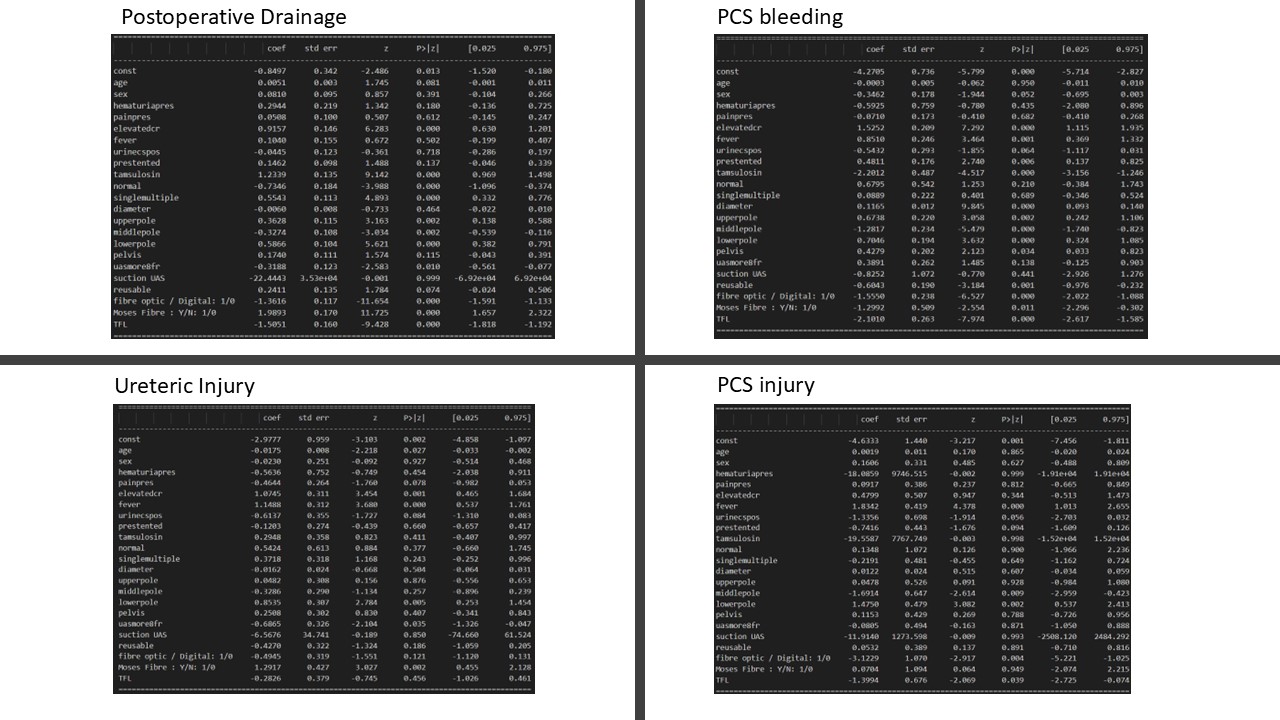


Figure 1. Logistic regression analysis for PCS bleeding, PCS injury, Ureteric Injury and Postoperative Drainage


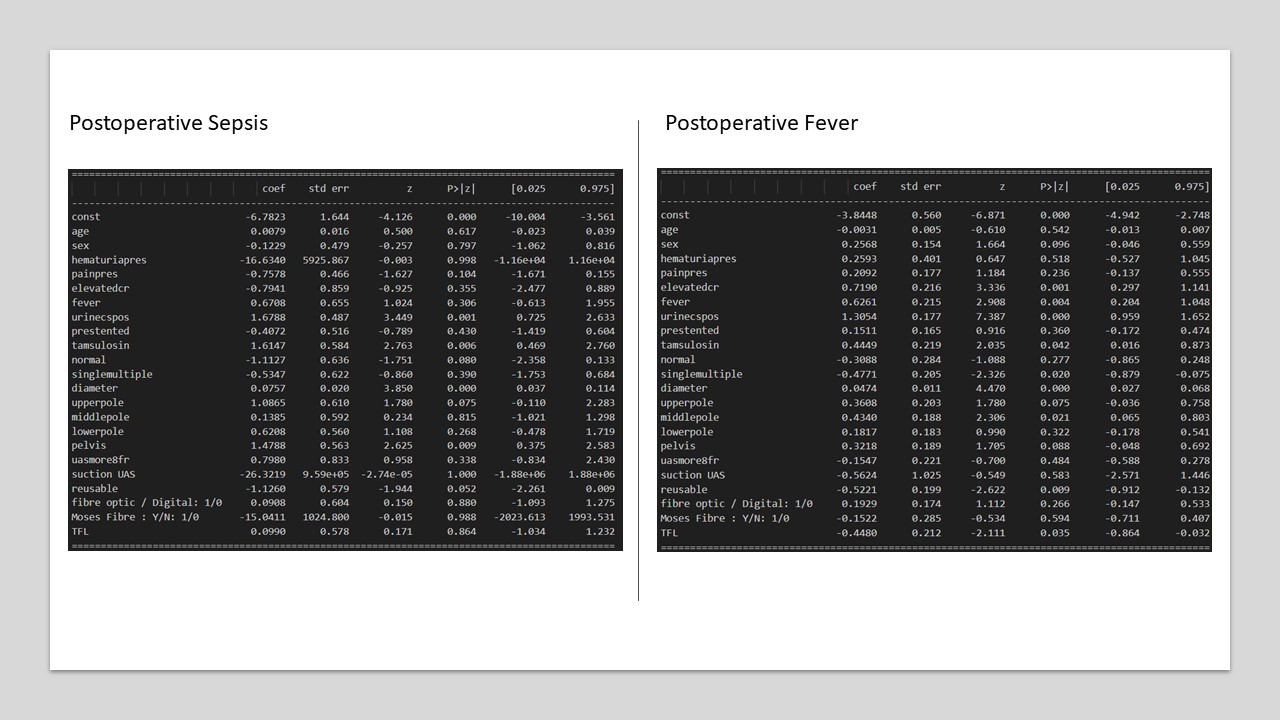


Figure 2. Logistic regression analysis for Postoperative Fever and Sepsis


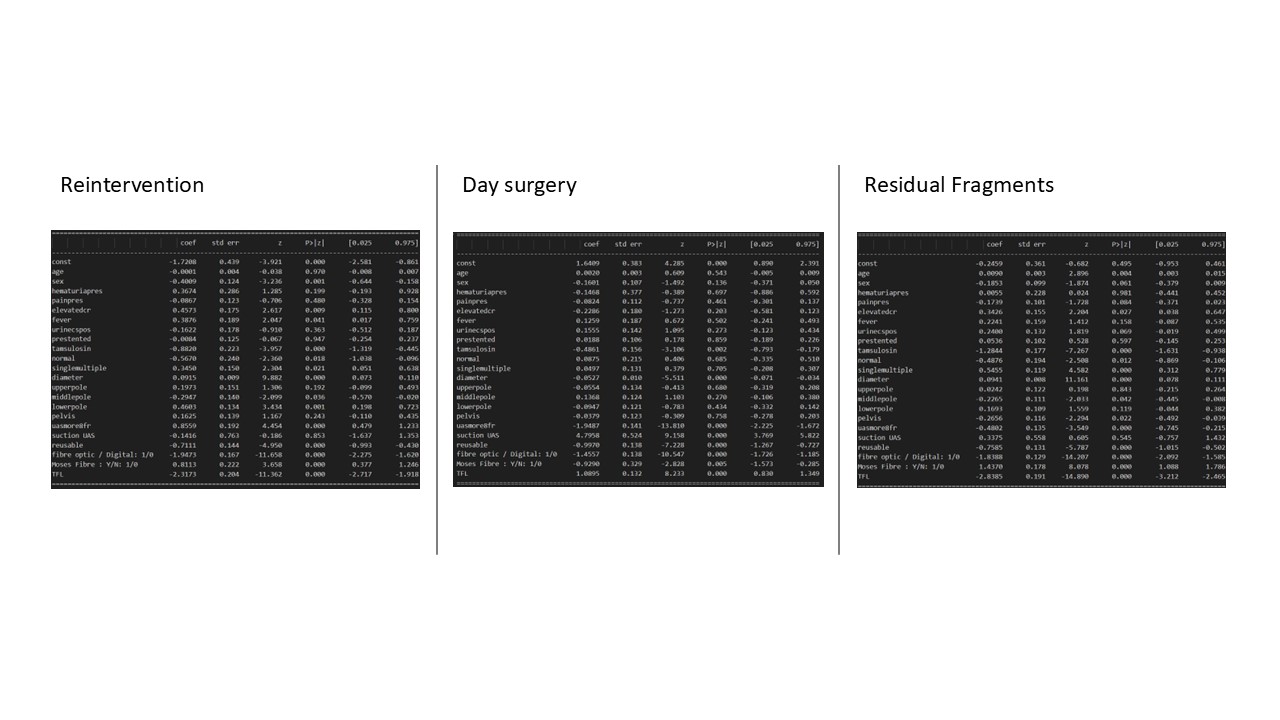


Figure 3. Logistic regression analysis for Day Surgery, Residual fragments and Reintervention

1. **EXPLAINABLE AI**


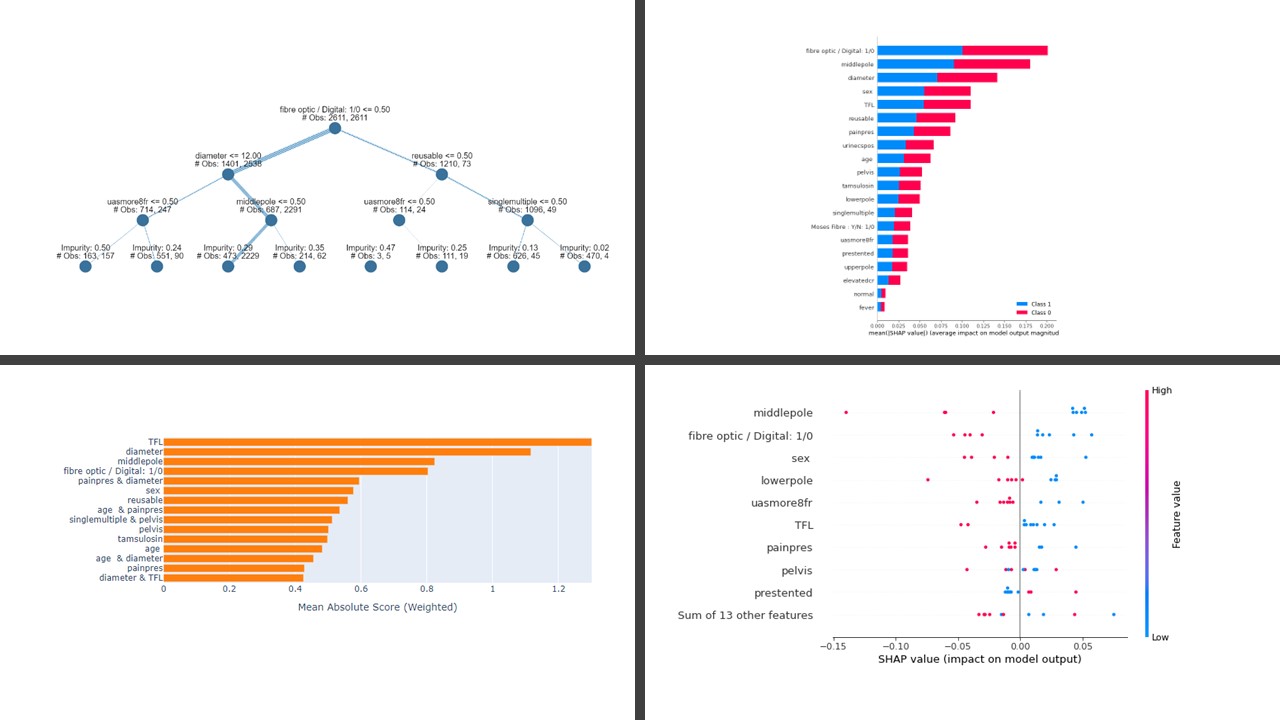


Figure 4. Explainable AI for PCS bleeding. Clockwise from the upper left figure: Explainable Tree, Feature Importance bar chart, Shap summary plot, Shap beeswarm chart


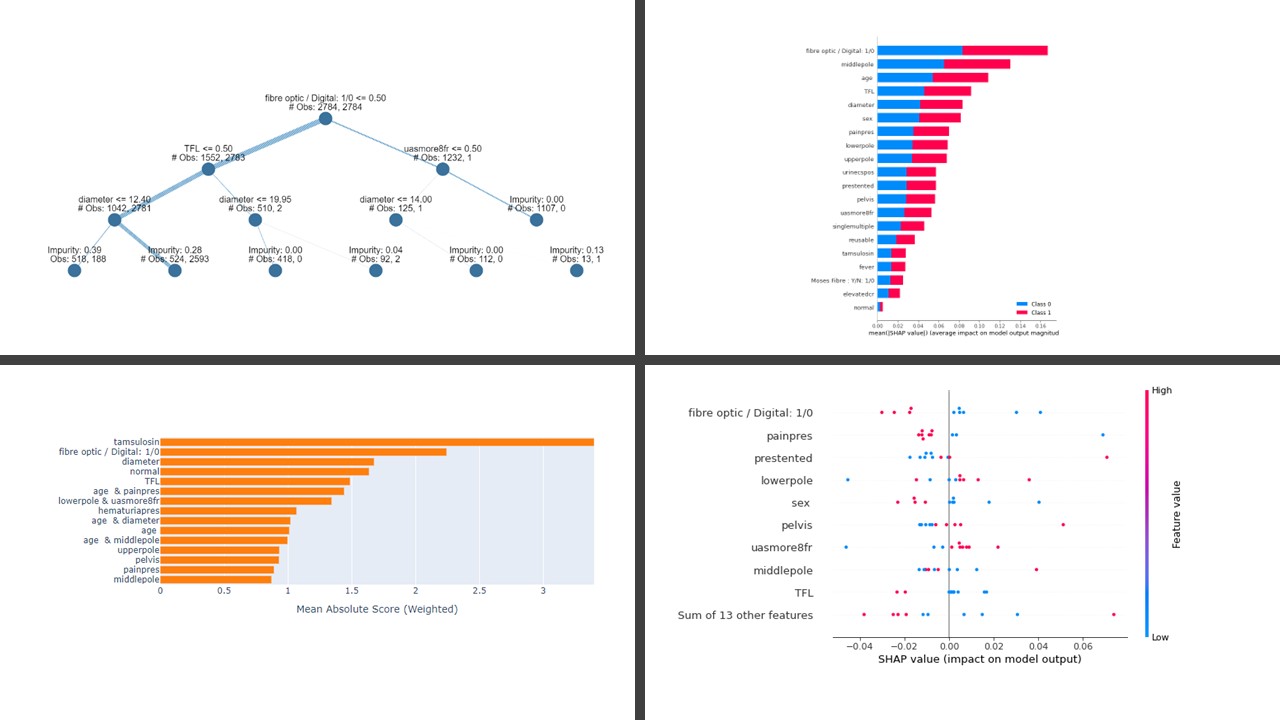


Figure 5. Explainable AI for PCS injury. Clockwise from the upper left figure: Explainable Tree, Feature Importance bar chart, Shap summary plot, Shap beeswarm chart


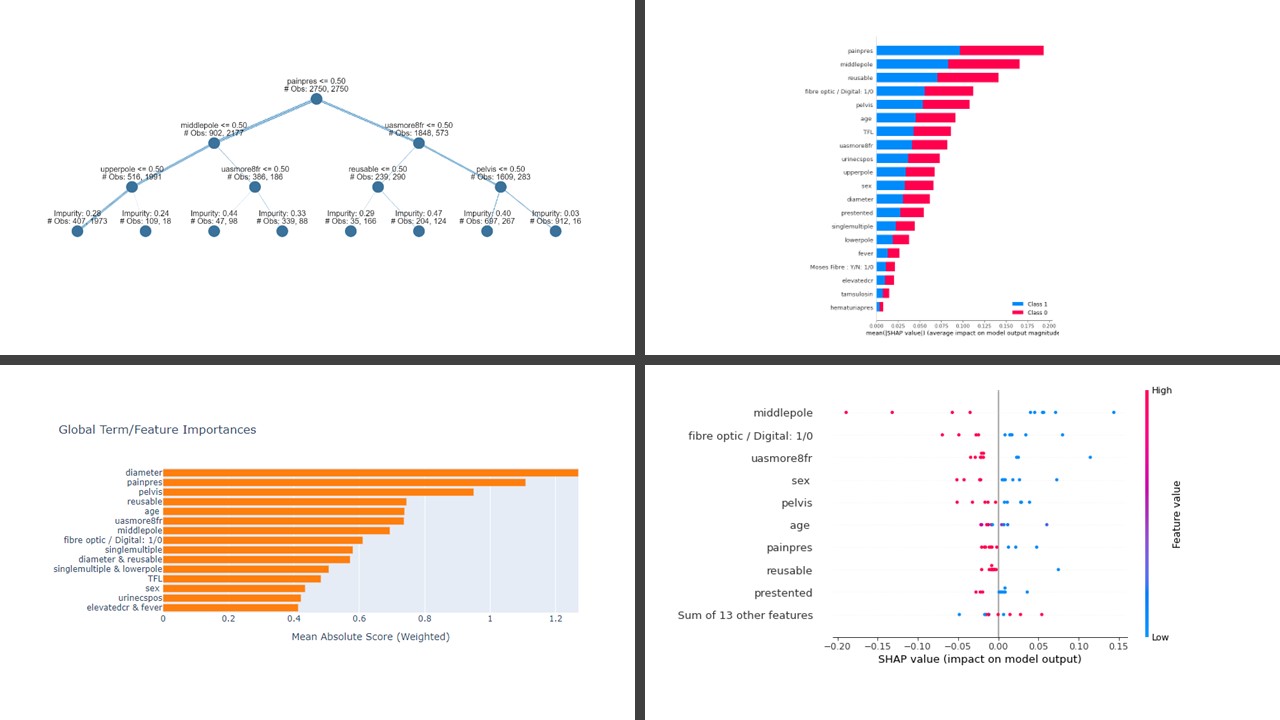


Figure 6. Explainable AI for ureteric injury. Clockwise from the upper left figure: Explainable Tree, Feature Importance bar chart, Shap summary plot, Shap beeswarm chart


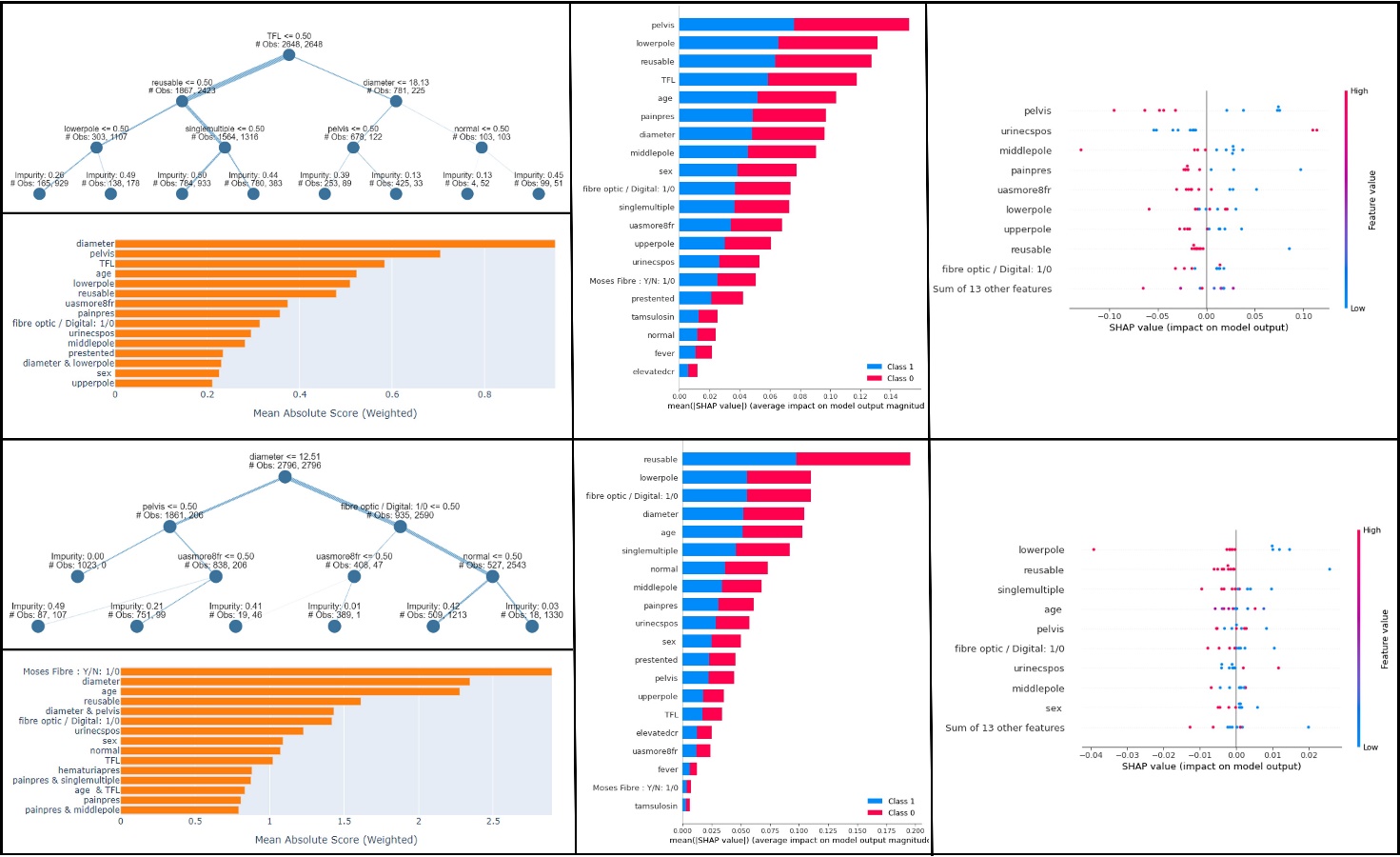


Figure 7. Explainable AI. In the upper half: explainable AI for prediction of postoperative fever (from the upper left: Explainable Tree, Feature Importance Plot, Shap Bar Chart, Chap Beeswarm Chart). In the lower half: explainable AI for prediction of postoperative sepsis (from the upper left: Explainable Tree, Feature Importance Plot, Shap Bar Chart, Chap Beeswarm Chart).


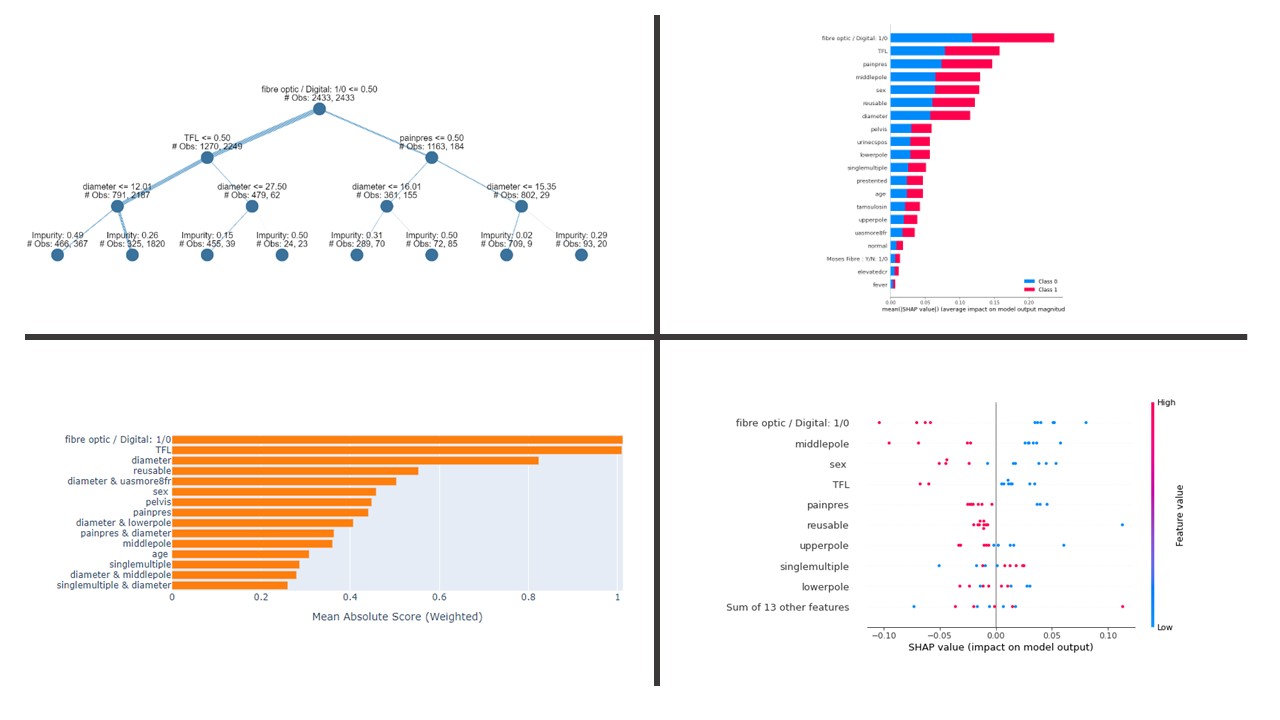


Figure 8. Explainable AI for reintervention. Clockwise from the upper left figure: Explainable Tree, Feature Importance bar chart, Shap summary plot, Shap beeswarm chart


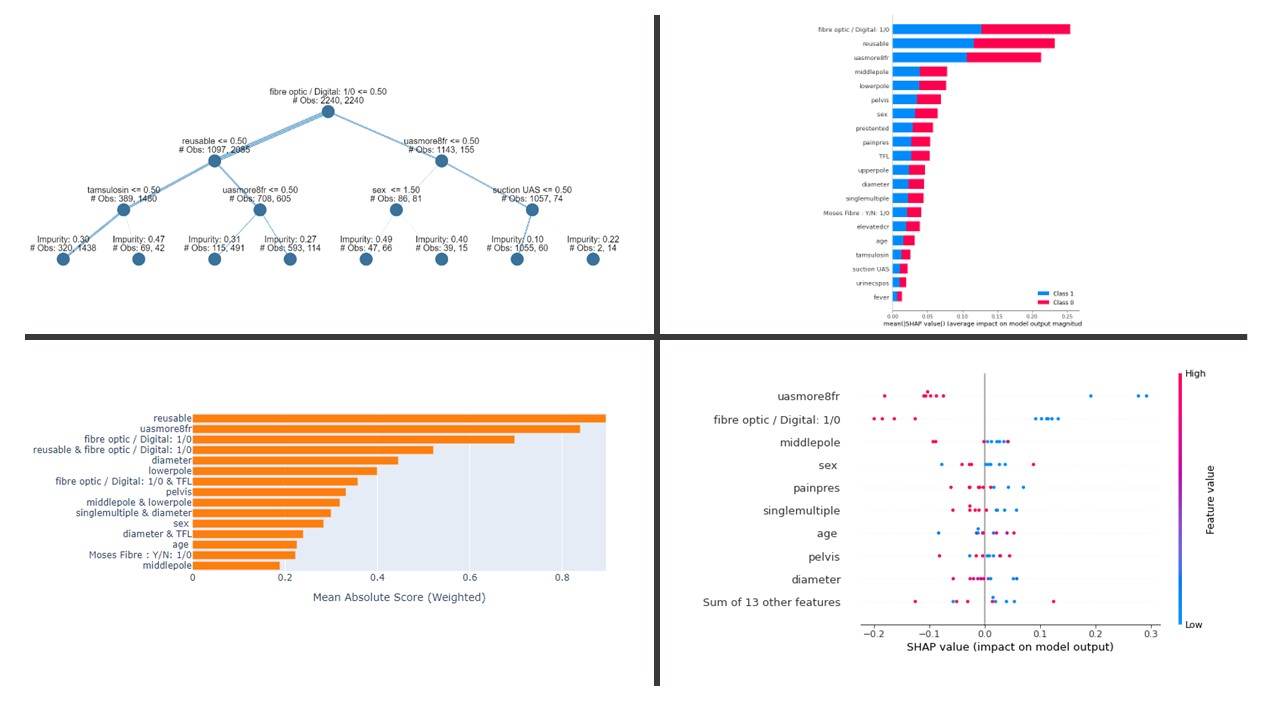


Figure 9. Explainable AI for same-day discharge. Clockwise from the upper left figure: Explainable Tree, Feature Importance bar chart, Shap summary plot, Shap beeswarm chart
